# Clinical Skill: The Ebbing Art of Medicine

**DOI:** 10.21315/mjms2021.28.1.13

**Published:** 2021-02-24

**Authors:** Ananda Datta

**Affiliations:** Department of Pulmonary Medicine and Critical Care, All India Institute of Medical Sciences, Bhubaneswar Odisha, India

**Keywords:** clinical skill, physical examination, history

## Abstract

Clinical history taking and physical examination are the essence of clinical medicine. However, the glare of modern diagnostic tools and techniques has overshadowed these basic but indispensable steps of diagnosis. Deterioration of clinical skills is a burning issue in this era due to over-reliance on high-end technology. Poor clinical judgment not only leads to mismanagement but also results in over-utilisation of health care resources. Moreover, with lesser time at the bedside, the physician-patient relationship is also getting compromised.

## Introduction

The advancement in medical technology in the last few decades has changed the approach towards the management of diseases. With the emerging knowledge in molecular pathophysiology and genetics the mysteries associated with the evolution of various diseases are being solved. High-tech laboratory investigations, latest diagnostic assays and innovative imaging tools have smoothened the path of diagnosis of various ailments. Newer therapeutic modalities are being discovered to curb the diseases that were once invincible. These enticing advancements have superseded the clinical skills of the physicians.

## Importance of Clinical Skill in Medicine

In medicine, each patient poses a clinical challenge to the treating physician. A competent physician should identify the clue hidden in the elaborated history and physical examination. He should also extract the key results from relevant investigations in order to reach a diagnostic conclusion. Studies have demonstrated that clinical history taking and physical examination play a major role in arriving at a diagnosis. In a prospective study, Peterson et al. (1) found that the history and physical examination led to the final diagnosis in 76% and 12% cases, respectively. On the other hand, laboratory investigations had a role in the diagnosis of only 10% cases. Another study concluded that medical history and physical examination guided to correct final diagnosis in 60%–70% instances. Even new diagnostic tests can lead to wrong results in 6%–9% cases (2). In a prospective study from India, it was found that 78.58% cases were diagnosed based on clinical history alone. Physical examination and investigations contributed to a diagnostic conclusion in only 8.17% and 13.27% cases, respectively (3).

Good history and a well-performed clinical examination can guide towards necessary investigation. Thus, both the patients and the health care system get relief from the burden of the ‘battery of tests.’ The laboratory abnormalities must be interpreted in an appropriate clinical context. The decisions made solely on the basis of printed laboratory results are invariably unproductive. A sound physician has the courage to knowingly overlook some laboratory data within the context of the specific clinical presentation.

History taking and physical examination foster and strengthen the patient-physician relationship by providing an opportunity for communication with the patients and allowing a touch of reassurance. This is essential not only for the extraction of valuable information that may be crucial for diagnosis but also to gain the confidence of patients to address the psychological part of the treatment. In a study analysing the relation between physician practice behaviour and patient satisfaction in outpatient services, Robbins et al. (4) concluded that patients were most satisfied when they were examined, provided health education and shared information about therapeutic interventions. Although some social scientific research demonstrated a negative impact of physical examination on patients, most of the medical literature favour it as a way of nurturing the physician-patient relationship (5).

## Reasons and Results of Poor Clinical Apt

Commercialisation of medicine and the introduction of consumerism in patient care have degraded the doctor-patient relationship. Modern medicine has become an organ-specific disease-centred approach rather than a holistic and humanistic approach. Bedside interactions have shifted to conference room, where the learning is based on the interpretation of the laboratory values and imaging results (6). A study showed that internal medicine interns are involved in direct patient care for less than 12% of their time (7).

There has been a constant degradation of clinical skills among physicians. Various reasons suggested are rushed rounds due to time constraints, lack of bedside teaching, lack of confidence in physical examination skills of medical educators, fear of demonstration of deficiencies in learners and over-reliance on laboratory data (8, 9, 10). In 1960s, 75% of the teaching time was spent near patient’s bedside (11). However, this has severely contracted to only 17% of teaching rounds in recent days (12). This results in hyposkilliac (13) physicians who lack the reasoning ability to assess the available information and create a suitable management plan. The physicians often rely largely on laboratory investigations and imaging for prompt diagnosis. Thus, the brain is gradually being replaced by the machine. A disease is being interpreted as a package of deranged laboratory values. But it has been well established that poor physical examination skills result in the injudicious use of diagnostic tests and mismanagement, leading to increased utilisation of health care resources. This is of great concern, especially in resource-limited settings. Inadequate physical examination resulted in missed or delayed diagnosis in 76% cases, misdiagnosis in 27%, unnecessary treatment in 18%, unnecessary diagnostic cost in 25% and unnecessary exposure to radiation or contrast in 17% of cases (14). A meta-analysis reported mean overutilisation rate of diagnostic testing to be 20.6% (15). Miyakis et al. (16) reported that 67.9% of laboratory tests ordered before any intervention were non-contributory in-patient management. Likewise, a single centre study from India has shown a similar proportion of unnecessary ordered tests (17). These unwise rampant usages of diagnostic tests create huge burden over a constrained economy (18). Oyedokun et al. (19) has re-emphasised the role of clinical skills in medical practice and healthcare in Africa.

## How to Enrich Clinical Skill?

Various literatures have discussed various methods to enhance clinical skills in medical practice in order to produce competent physicians. Practice makes a man perfect. It is needless to say that, for improvement of skill, more time has to be spent at patient’s bedside by both the trainer and the trainee. In addition to the traditional ways of bedside teaching, various structured models for development of clinical skills have been proposed. Clinical skill laboratory can provide adequate facility to acquire skills during early phase of medical training (20). Simulation based learning involves an artificial way to replace a real patient, allowing experimental learning in a deliberate way (21). Also, Peyton’s four step approach involves training under direct observation with feedback from trainer (22). Problem based learning promotes efficient knowledge development, critical thought process and communication skills (23). Structured clinical observation has been shown to improve clinical skills in limited-time settings (24). Also, the training of faculty in clinical teaching methods would be beneficial in bedside teaching (25).

## Conclusion

However, it should also be kept in mind that an overconfident clinician may sometimes misdiagnose relying on clinical findings only, wherein a few requisite high-tech investigation could have led a proper diagnosis. So, it is imperative not to overemphasis on artificially intelligent medicine but to use the compassionate clinical skills with wise use of diagnostic assays to solve clinical puzzles.
